# Vonoprazan vs Proton Pump Inhibitors for Gastroprotection in Anticoagulated Patients: A Real-World Cohort Study

**DOI:** 10.7759/cureus.108039

**Published:** 2026-04-30

**Authors:** Hatem Ahmed, Imad Alabdul Razzak, Eyad Abdulrazzak, Sameh Gomaa, Khloud Abdelrazeq, Annapurna Korimilli, Risha Khetarpal, Motaz Almahmood, Aamir Shahzad, Khaled Elsokary

**Affiliations:** 1 Department of Internal Medicine, Phoenixville Hospital, Phoenixville, USA; 2 Department of Hepatology, Beth Israel Deaconess Medical Center, Boston, USA; 3 Faculty of Medicine, Cairo University, Cairo, EGY; 4 Department of Gastroenterology, Penn Gastroenterology Limerick, Limerick, USA; 5 Department of Internal Medicine, Tower Health Medical Group, Douglassville, USA; 6 Department of Gastroenterology, Lehigh Valley Health Network, Allentown, USA; 7 Department of Gastroenterology, Reading Hospital - Tower Health, West Reading, USA

**Keywords:** direct-acting oral anticoagulants, gastrointestinal bleeding, gastroprotection, potassium-competitive acid blocker, proton pump inhibitor, therapeutic anticoagulation, vonoprazan

## Abstract

Introduction

Oral anticoagulation is associated with an increased risk of GI bleeding. Proton pump inhibitors (PPIs) are commonly used for gastroprotection but have pharmacologic limitations. This study examined whether vonoprazan is associated with different one-year bleeding and clinical outcomes compared with PPIs among anticoagulated adults.

Methods

We conducted a retrospective cohort study using the TriNetX database to identify adults receiving oral anticoagulants who initiated vonoprazan or a PPI between 2015 and 2025. Propensity score matching (1:1) was performed to balance demographics, comorbidities, procedures, concomitant medications, and baseline laboratory values. Outcomes were assessed one year after treatment initiation. The primary outcome was GI bleeding; secondary outcomes included all-cause mortality, blood transfusion, and hemoglobin level.

Results

After matching, 3,107 patients were included in each group. GI bleeding was lower with vonoprazan than with PPIs (55 (1.96%) vs 88 (3.18%); OR 0.61; 95% CI 0.43-0.86). All-cause mortality (177 (5.70%) vs 290 (9.38%); OR 0.58; 95% CI 0.48-0.71) and blood transfusion (≤10 (0.33%) vs 27 (0.88%); OR 0.37; 95% CI 0.18-0.76) were also lower with vonoprazan. Mean hemoglobin at one year was modestly higher with vonoprazan (11.76 ± 2.13 vs 11.54 ± 2.34 g/dL).

Conclusions

In a propensity-matched real-world anticoagulated cohort, vonoprazan use was associated with lower one-year GI bleeding and transfusion rates, with modestly higher hemoglobin levels, compared with PPIs. These findings are hypothesis-generating and warrant confirmation in prospective studies.

## Introduction

Oral anticoagulants, including vitamin K antagonists (e.g., warfarin) and direct oral anticoagulants (DOACs), are foundational for managing thromboembolic conditions such as atrial fibrillation, venous thromboembolism, and cardiovascular disease. However, they carry a measurable risk of GI bleeding: large population data estimate a GI bleeding incidence of approximately 12.6 per 1,000 person-years among DOAC users [1]. Similarly, a population-based cohort study reported an incidence of 3.1 GI bleeding events per 100 person-years among patients receiving warfarin therapy [2].

Proton pump inhibitors (PPIs) have long served as the conventional gastroprotective strategy in anticoagulated patients, especially those deemed at high risk for GI bleeding [3]. Yet, PPIs possess several pharmacologic limitations: they require acid activation, have a delayed onset to maximal acid suppression, and demonstrate considerable interindividual variability, much of which is influenced by CYP2C19 polymorphisms and potential drug-drug interactions [4,5]. Moreover, PPI effectiveness depends on timing in relation to meals, and maximal efficacy typically develops over several days of use [5,6].

In contrast, potassium-competitive acid blockers (P-CABs) such as vonoprazan provide rapid, potent, and sustained inhibition of the gastric H⁺/K⁺-ATPase by reversibly blocking the K⁺ binding site [7,8]. This mechanism allows for acid suppression that is faster and more consistent than that achieved with PPIs, largely independent of acid activation or CYP2C19 genotype [7,8]. Vonoprazan’s acid-stable profile and minimal influence from food intake offer theoretical advantages in maintaining intragastric pH stability [8,9].

Vonoprazan has shown superior or comparable effects vs PPIs in several acid-related contexts, including erosive esophagitis and prevention of rebleeding in high-risk peptic ulcer disease and upper GI bleeding [10-13]. However, most comparative studies have been limited to patients on antiplatelet agents or those undergoing advanced endoscopic procedures, with limited data directly focusing on anticoagulated populations [14-17].

To our knowledge, no prior studies have exclusively compared vonoprazan and PPIs among patients receiving oral anticoagulation therapy, irrespective of concomitant antiplatelet use. This represents a critical evidence gap, particularly given the rising use of DOACs and the growing availability of P-CABs such as vonoprazan. We therefore conducted a multicenter, real-world, propensity score-matched analysis using the TriNetX research network to compare one-year GI bleeding incidence, mortality, and related outcomes among anticoagulated patients prescribed vonoprazan vs those prescribed PPIs.

An abstract related to this work was presented as a poster at the American College of Gastroenterology (ACG) Annual Scientific Meeting (Phoenix, Arizona, USA; October 24-29, 2025) and was published in the ACG conference supplement.

## Materials and methods

Study design and data source

This retrospective cohort study was conducted using TriNetX, a global federated research network that provides real-time access to de-identified electronic health records (EHRs) from more than 250 million patients across multiple healthcare organizations worldwide. For this analysis, we utilized the Global Collaborative Network, a subset of TriNetX optimized for multicenter clinical research.

The TriNetX platform provides structured clinical data extracted from participating healthcare systems using natural language processing and standardized medical terminologies. Diagnoses, procedures, medications, and laboratory data are coded according to the International Classification of Diseases, 10th Revision (ICD-10), Current Procedural Terminology (CPT), RxNorm, and Logical Observation Identifiers Names and Codes (LOINC) standards.

Study population and cohort definition

A real-time query was conducted within the Global Collaborative Network on the TriNetX platform to identify adult patients (aged ≥18 years) who received oral anticoagulation therapy, identified through relevant RxNorm codes for warfarin, dabigatran, rivaroxaban, apixaban, or edoxaban.

Patients were classified into two cohorts: (1) Vonoprazan Group: patients who were prescribed vonoprazan after initiating anticoagulation therapy and (2) PPI Group: patients who were prescribed a PPI after starting anticoagulation and had no recorded vonoprazan exposure.

The study period spanned from January 1, 2015, corresponding to the earliest reported clinical use of vonoprazan [18], through the date of database query (September 10, 2025). The index date was defined as the first prescription of vonoprazan (vonoprazan group) or the first prescription of a PPI (PPI group) occurring after initiation of oral anticoagulation.

A complete list of all ICD, RxNorm, and CPT codes used for cohort selection and outcome definition is provided in Appendix A.

Covariates and baseline characteristics

Covariates were extracted from each patient’s five-year medical history prior to the index date to account for potential confounders. These included demographics such as age, sex, and race; comorbidities including history of thromboembolism, ischemic heart disease, cerebral infarction, type 2 diabetes mellitus, hypertension, heart failure, and chronic kidney disease; procedures including esophagogastroduodenoscopy and colonoscopy; medications including use of antiplatelet agents and nonsteroidal anti-inflammatory drugs (NSAIDs); and baseline laboratory values such as hemoglobin, platelet count, and international normalized ratio (INR).

Study outcomes

The primary outcome was the incidence of GI bleeding within one year of the index date, defined by diagnostic codes for melena, hematemesis, or unspecified GI hemorrhage. Secondary outcomes included all-cause mortality at one year, occurrence of blood transfusion events, and mean hemoglobin levels at one year of follow-up. Cause-specific mortality and hospitalization data were not available due to inherent limitations of the TriNetX platform. Patients with any recorded outcome of interest prior to their index date were excluded from the analysis for that specific outcome but remained eligible for matching in the analysis of other outcomes.

Statistical analysis

The study size was determined by all eligible TriNetX patients meeting the inclusion criteria during the study period. All analyses were conducted within the TriNetX analytics environment using built-in modules (TriNetX, LLC, Cambridge, MA, USA). To reduce confounding, 1:1 propensity score matching was performed using greedy nearest-neighbor matching without replacement and a caliper width of 0.1 pooled SDs. Propensity scores were estimated via logistic regression incorporating prespecified baseline characteristics. Balance after matching was evaluated using standardized mean differences (SMDs), with an absolute SMD <0.1 indicating acceptable balance.

Binary outcomes within each follow-up window were evaluated using TriNetX Measure of Association analyses, reporting ORs with 95% CIs. For each specific outcome, patients with that outcome documented prior to the start of the follow-up window were excluded from the corresponding outcome analysis while remaining eligible for analyses of other outcomes. Laboratory outcomes were compared between cohorts using t-tests among patients with available results; no imputation was performed. All tests were two-sided, and p-values <0.05 were considered statistically significant.

Ethics approval

This retrospective study used de-identified EHR data. The study was reviewed and deemed exempt by the local Institutional Review Board. All procedures were conducted in accordance with the ethical standards of the institutional and national research committees, the 1964 Declaration of Helsinki and its later amendments, and the Declaration of Taipei for health databases.

## Results

Cohort selection

A total of 3,158 patients who received vonoprazan and 1,767,440 patients who received a PPI after anticoagulation were initially identified. After applying inclusion and exclusion criteria and performing 1:1 propensity score matching, 3,107 patients remained in each group for final analysis.

Baseline characteristics

Before propensity score matching, there were notable differences between the vonoprazan and PPI cohorts. Comorbidities showed divergent patterns before matching. Chronic ischemic heart disease was more prevalent in the PPI cohort (513,356 (29.0%) vs 370 (11.7%); SMD = 0.44), whereas gastroesophageal reflux disease was more common in the vonoprazan cohort (1,644 (52.1%) vs 510,315 (28.9%); SMD = 0.49). Vonoprazan patients also had higher rates of gastritis/duodenitis (675 (21.4%) vs 106,353 (6.0%)) and gastric ulcer (544 (17.2%) vs 35,576 (2.0%)).

Medication exposure also differed: NSAID use was higher in the vonoprazan cohort (1,839 (58.2%) vs 751,579 (42.5%)), while warfarin (311,951 (17.6%) vs 353 (11.2%)) and heparin (663,343 (37.5%) vs 655 (20.7%)) were more common in the PPI cohort. After 1:1 matching, all baseline variables were well balanced (all SMDs < 0.10).

Full baseline characteristics before and after matching are presented in Table 1. All outcome comparisons were performed in the matched cohorts.

**Table 1:** Baseline characteristics of patients in the vonoprazan cohort and PPI cohort before and after propensity score matching NSAIDs, nonsteroidal anti-inflammatory drugs; PPI, proton pump inhibitor; SMD, standardized mean difference

Characteristic	Before propensity score matching			After propensity score matching		
	Vonoprazan (n = 3,158)	PPI (n = 1,767,440)	SMD	Vonoprazan (n = 3,107)	PPI (n = 3,107)	SMD
Demographics						
Age at index, mean ± SD	75.9 ± 11.6	68.8 ± 14.1	0.551	75.8 ± 11.6	76.1 ± 11.4	0.024
Male, n (%)	1,495 (47.3)	882,543 (49.9)	0.052	1,480 (47.6)	1,466 (47.2)	0.009
Female, n (%)	1,657 (52.5)	834,406 (47.2)	0.105	1,621 (52.2)	1,629 (52.4)	0.005
Race/ethnicity, n (%)						
Caucasian	374 (11.8)	1,182,852 (66.9)	1.365	374 (12.0)	396 (12.7)	0.021
African American	1,956 (61.9)	685,808 (38.8)	0.476	1,910 (61.5)	1,902 (61.2)	0.005
Hispanic/Latino	42 (1.3)	67,734 (3.8)	0.158	42 (1.4)	43 (1.4)	0.003
Asian	14 (0.4)	59,235 (3.4)	0.214	14 (0.5)	10 (0.3)	0.021
Comorbidities, n (%)						
Atrial fibrillation/flutter	1,503 (47.6)	733,114 (41.5)	0.123	1,476 (47.5)	1,532 (49.3)	0.036
Venous thrombosis	132 (4.2)	256,344 (14.5)	0.36	132 (4.2)	119 (3.8)	0.021
Pulmonary embolism	121 (3.8)	179,690 (10.2)	0.25	121 (3.9)	105 (3.4)	0.028
Prosthetic heart valve	61 (1.9)	67,704 (3.8)	0.114	61 (2.0)	52 (1.7)	0.022
Essential hypertension	1,581 (50.1)	965,180 (54.6)	0.091	1,555 (50.0)	1,537 (49.5)	0.012
Diabetes mellitus	795 (25.2)	484,283 (27.4)	0.051	781 (25.1)	741 (23.8)	0.03
Chronic kidney disease	384 (12.2)	360,742 (20.4)	0.225	381 (12.3)	403 (13.0)	0.021
Cirrhosis of the liver	58 (1.8)	44,007 (2.5)	0.045	58 (1.9)	56 (1.8)	0.005
Cerebral infarction	534 (16.9)	150,495 (8.5)	0.254	521 (16.8)	538 (17.3)	0.015
Transient ischemic attacks	53 (1.7)	62,592 (3.5)	0.117	53 (1.7)	57 (1.8)	0.01
Chronic ischemic heart disease	370 (11.7)	513,356 (29.0)	0.44	370 (11.9)	362 (11.7)	0.008
Heart failure	1,068 (33.8)	471,811 (26.7)	0.156	1,046 (33.7)	1,075 (34.6)	0.02
Gastric ulcer	544 (17.2)	35,576 (2.0)	0.534	512 (16.5)	507 (16.3)	0.004
Duodenal ulcer	72 (2.3)	19,413 (1.1)	0.092	72 (2.3)	78 (2.5)	0.013
Peptic ulcer	31 (1.0)	19,172 (1.1)	0.01	31 (1.0)	30 (1.0)	0.003
Gastritis/duodenitis	675 (21.4)	106,353 (6.0)	0.458	636 (20.5)	627 (20.2)	0.007
Esophagitis	112 (3.5)	36,098 (2.0)	0.091	112 (3.6)	119 (3.8)	0.012
Gastroesophageal reflux disease	1,644 (52.1)	510,315 (28.9)	0.486	1,598 (51.4)	1,615 (52.0)	0.011
Procedures, n (%)						
Esophagogastroduodenoscopy	63 (2.0)	206,405 (11.7)	0.391	63 (2.0)	63 (2.0)	0.001
Colonoscopy	266 (8.4)	134,475 (7.6)	0.03	266 (8.6)	295 (9.5)	0.033
Medications, n (%)						
Warfarin	353 (11.2)	311,951 (17.6)	0.185	350 (11.3)	328 (10.6)	0.023
Dabigatran	57 (1.8)	32,048 (1.8)	0.001	56 (1.8)	38 (1.2)	0.047
Heparin	655 (20.7)	663,343 (37.5)	0.376	644 (20.7)	626 (20.1)	0.014
Edoxaban	859 (27.2)	11,692 (0.7)	0.83	810 (26.1)	842 (27.1)	0.023
Apixaban	606 (19.2)	559,818 (31.7)	0.29	604 (19.4)	537 (17.3)	0.056
Rivaroxaban	396 (12.5)	256,779 (14.5)	0.058	395 (12.7)	381 (12.3)	0.014
Enoxaparin	224 (7.1)	512,777 (29.0)	0.595	224 (7.2)	217 (7.0)	0.009
NSAIDs	1,839 (58.2)	751,579 (42.5)	0.318	1,788 (57.5)	1,698 (54.7)	0.058
Platelet aggregation inhibitors	121 (3.8)	214,477 (12.1)	0.31	121 (3.9)	127 (4.1)	0.01
Laboratory, mean ± SD						
Hemoglobin	12.0 ± 2.3	11.9 ± 2.5	0.038	12.0 ± 2.3	11.9 ± 2.3	0.026
Platelets	228.2 ± 89.2	236.7 ± 102.6	0.088	228.2 ± 89.3	231.1 ± 93.7	0.032
International normalized ratio	1.2 ± 0.4	1.4 ± 0.8	0.359	1.2 ± 0.4	1.2 ± 0.5	0.085

Primary and secondary outcomes

At one year, patients prescribed vonoprazan experienced fewer GI bleeding events than those receiving PPIs, with incidences of 55 (1.96%) and 88 (3.18%), respectively (OR 0.61; 95% CI, 0.43-0.86), corresponding to an absolute risk reduction of 1.22% (Table 2).

**Table 2:** One-year clinical outcomes in matched vonoprazan vs PPI cohorts p-Values <0.05 were considered statistically significant. Significant p-values are marked with an asterisk (*). Counts ≤10 are reported as “≤10” in accordance with TriNetX data privacy policies. PPI, proton pump inhibitor

Outcome	Vonoprazan	PPI	OR	95% CI	p-Value
Primary outcomes, n (%)					
GI bleeding	55 (1.96)	88 (3.18)	0.61	0.43-0.86	0.0039^*^
Secondary outcomes, n (%)					
All-cause mortality	177 (5.7)	290 (9.38)	0.58	0.48-0.71	<0.0001^*^
Blood transfusion	≤10 (0.326)	27 (0.883)	0.37	0.18-0.76	0.0048^*^
Tertiary outcome, mean ± SD					
Hemoglobin (g/dL)	11.756 ± 2.132	11.537 ± 2.339	t = 2.993		0.0006^*^

All-cause mortality at one year was significantly lower in the vonoprazan group than in the PPI group (177 (5.70%) vs 290 (9.38%); OR 0.58; 95% CI, 0.48-0.71; p < 0.0001). Given the likelihood of residual confounding and the absence of cause-specific mortality data, this result should be interpreted cautiously.

Blood transfusion was less frequent in patients prescribed vonoprazan than in those on PPIs (≤10 (0.33%) vs 27 (0.88%); OR 0.37; 95% CI, 0.18-0.76; p = 0.0048). Mean hemoglobin levels at one-year follow-up were modestly higher in the vonoprazan group than in the PPI group (11.76 ± 2.13 g/dL vs 11.54 ± 2.34 g/dL; p = 0.0006).

## Discussion

In this real-world, propensity-matched cohort of anticoagulated patients, vonoprazan use was associated with a lower one-year risk of GI bleeding compared with PPIs, along with lower all-cause mortality, fewer transfusion events, and slightly higher hemoglobin levels. Together, these findings suggest that vonoprazan may be associated with lower GI bleeding outcomes compared to traditional PPIs in patients receiving oral anticoagulation. However, given the observational design and code-based outcomes, these findings should be interpreted as associations and require prospective confirmation.

GI bleeding is a well-recognized complication of antithrombotic therapy, including both antiplatelet and anticoagulant therapies, with risk increasing as treatment intensity escalates. Data suggest approximately a 1.8-fold increase in bleeding with aspirin and a 7-fold increase with dual antiplatelet therapy [19], while among patients on DOACs, major GI bleeding occurs in roughly two to three events per 100 person-years, depending on the specific agent and underlying risk profile [1,20]. These observations highlight the importance of reliable gastroprotective strategies in high-risk populations.

PPIs have long served as the mainstay of GI protection among high-risk patients receiving anticoagulants. In warfarin users, PPI co-therapy has been linked to fewer hospitalizations for GI bleeding [21]. However, shared cytochrome P450 metabolic pathways between warfarin and PPIs can affect INR stability, necessitating more frequent monitoring [22]. In the context of DOACs, observational data suggest PPI co-therapy is associated with a lower risk of upper GI bleeding across anticoagulant agents [21].

Despite their widespread use, PPIs are not without limitations. Their pharmacologic profile includes a delayed onset of action, a requirement for meal-timed dosing, and considerable interindividual variability, much of which is attributed to CYP2C19 polymorphisms and drug-drug interactions [4-6]. These factors can lead to unpredictable acid suppression in real-world settings, particularly when immediate and consistent protection is desired.

Vonoprazan, a P-CAB, has emerged as a potent and rapidly acting alternative to traditional PPIs. Its pharmacologic profile includes acid stability, independence from food for activation, and more uniform efficacy across CYP2C19 genotypes [7-9]. It achieves near-neutral gastric pH within hours of administration and maintains potent acid suppression for over 24 hours with once-daily dosing [9]. Combined, these characteristics make vonoprazan an attractive option for acid suppression in high-risk patients, including those on anticoagulation therapy. Mechanistically, this rapid and sustained intragastric pH control could improve clot stability and mucosal hemostasis in the upper GI tract, thereby reducing recurrent bleeding from acid-exposed lesions. While this proposed mechanism remains speculative in the context of this database study (as lesion location, endoscopic stigmata, and pH metrics were unavailable), the biological plausibility supports further prospective evaluation in anticoagulated populations.

Nevertheless, vonoprazan’s use requires caution due to potential drug-drug interactions. In vitro studies have demonstrated inhibitory activity on several cytochrome P450 enzymes, including CYP3A4, CYP2C9, CYP2D6, and CYP2B6 [23]. Clinical implications include potential attenuation of the antiplatelet effects of clopidogrel and prasugrel [24] and elevated tacrolimus levels in transplant recipients [25], necessitating careful consideration of concomitant medications.

To date, most comparative investigations between vonoprazan and PPIs have been conducted in populations treated with antiplatelet agents or in postprocedural contexts. For example, a large Japanese study demonstrated reduced bleeding and readmission rates following gastric endoscopic submucosal dissection (ESD) in patients treated with vonoprazan [16]. Another study reported a ~30% reduction in delayed bleeding after gastroduodenal ESD with vonoprazan vs PPIs [17]. Several randomized trials and observational studies in patients with high-risk peptic ulcer bleeding after successful endoscopic hemostasis have found vonoprazan to be comparable or noninferior to PPIs with respect to rebleeding and mortality [11-13], while other work has evaluated ischemic heart disease patients receiving dual or multiple antithrombotic agents [15].

However, none of these studies specifically targeted patients exclusively on oral anticoagulants, a group with distinct bleeding patterns and pharmacologic considerations. Our study addresses this critical gap by evaluating the comparative safety and effectiveness of vonoprazan vs PPIs in a large, real-world cohort of anticoagulated patients using a rigorously matched propensity score design.

In the United States, vonoprazan is approved for indications such as treatment and maintenance of healing of erosive esophagitis and *Helicobacter pylori *eradication, but it is not currently labeled for prevention of GI bleeding or for routine gastroprotection in anticoagulated patients. Accordingly, the coadministration of vonoprazan with oral anticoagulants evaluated in this study represents an off-label use, and our findings should be interpreted as hypothesis-generating rather than as regulatory or prescribing guidance.

Limitations

This study has several limitations inherent to its retrospective, observational design using a federated EHR network. First, despite 1:1 propensity score matching, residual and unmeasured confounding cannot be excluded. We lacked granular data on several important determinants of GI bleeding risk (e.g., alcohol use and over-the-counter aspirin/NSAIDs), as well as prescriber preference and disease severity cues that can drive treatment selection (channeling/indication bias). Socioeconomic factors and formulary access may influence the selection of vonoprazan vs PPIs and were not directly measured.

Second, exposure misclassification is possible. We could not verify medication adherence, formulation, or persistence. Furthermore, a washout period and time-varying exposure modeling were not applied; consequently, prior acid-suppressive therapy, temporary discontinuations, and treatment crossover (e.g., between PPIs and vonoprazan) could not be fully characterized. For warfarin users, time in the therapeutic range and longitudinal INR values were unavailable, limiting adjustment for anticoagulation control.

Third, outcome misclassification may occur because events were identified by diagnostic codes without systematic endoscopic confirmation or bleeding severity grading. Diagnosis codes may capture both upper and lower GI bleeding, and “unspecified GI hemorrhage” can reflect heterogeneous bleeding sources. Additionally, we were unable to assess cause-specific mortality and GI-related hospitalizations due to platform constraints.

Finally, generalizability may be limited. The matched cohorts skewed older and reflect the practice patterns of participating TriNetX organizations and the timeline of vonoprazan availability; findings may not extrapolate to younger populations, nonparticipating centers, or regions with different prescribing habits. Moreover, all-cause mortality is highly vulnerable to confounding in EHR-based observational studies, and cause-specific mortality was not available; therefore, mortality findings cannot be definitively attributed to acid suppression.

## Conclusions

In this propensity-matched real-world cohort of patients receiving oral anticoagulation, vonoprazan use was associated with a lower one-year risk of coded GI bleeding compared with PPIs, along with fewer transfusions and modestly higher hemoglobin levels. To our knowledge, this is the first study to directly compare vonoprazan vs PPIs in an anticoagulated population, addressing an important evidence gap beyond antiplatelet and postprocedural settings.

Given the retrospective design and the potential for residual confounding, these associations should be interpreted as hypothesis-generating and cannot establish causality, particularly for all-cause mortality. Prospective randomized trials and pragmatic effectiveness studies stratified by anticoagulant class and dose are needed to define the role of vonoprazan for gastroprotection in anticoagulated patients.

## Appendices

Appendix A 

**Table 3:** List of ICD-10, ICD-9, RxNorm, and CPT codes used in cohort identification and outcome definition

Description	Code
Inclusion criteria	
Adults ≥ 18 years	Demographics: Age ≥ 18 years
Anticoagulation (any of the following ≥ January 1, 2015)	
Dabigatran etexilate	RxNorm: 1037042
Rivaroxaban	RxNorm: 1114195
Warfarin	RxNorm: 11289
Apixaban	RxNorm: 1364430
Edoxaban	RxNorm: 1599538
Exposure definitions	
Vonoprazan (study drug)	RxNorm: 2604577
Proton pump inhibitors (comparator group)	ATC: A02BC
Outcome definitions	
Gastrointestinal bleeding	
Hematemesis	ICD-10: K92.0
Melena	ICD-10: K92.1
Gastrointestinal hemorrhage, unspecified	ICD-10: K92.2
Gastrointestinal hemorrhage (unspecified type)	ICD-9: 578
Mortality	Demographics: Deceased
Blood transfusion	CPT: 36430 - transfusion, blood or blood components
Hemoglobin (mass/volume) in blood	TriNetX lab code: 9014
Comorbidities and baseline variables (propensity score matching)	
Atrial fibrillation and flutter	ICD-10: I48
Other venous embolism and thrombosis	ICD-10: I82
Pulmonary embolism	ICD-10: I26
Presence of prosthetic heart valve	ICD-10: Z95.2
Essential (primary) hypertension	ICD-10: I10
Diabetes mellitus (types E08–E13)	ICD-10: E08–E13
Chronic kidney disease (CKD)	ICD-10: N18
Fibrosis and cirrhosis of the liver	ICD-10: K74
Cerebral infarction	ICD-10: I63
Transient cerebral ischemic attacks and related syndromes	ICD-10: G45
Chronic ischemic heart disease	ICD-10: I25
Heart failure	ICD-10: I50
Gastric ulcer	ICD-10: K25
Duodenal ulcer	ICD-10: K26
Peptic ulcer, site unspecified	ICD-10: K27
Gastritis and duodenitis	ICD-10: K29
Esophagitis	ICD-10: K20
Gastro-esophageal reflux disease	ICD-10: K21
Procedures (pre-index screening)	
Esophagogastroduodenoscopy	CPT: 1021431
Colonoscopy, flexible	CPT: 1022231
Additional medication variables	
Heparin	RxNorm: 5224
Enoxaparin	RxNorm: 67108
Non-steroidal anti-inflammatory analgesics	VA Code: CN104
Platelet aggregation inhibitors	VA Code: BL117
Laboratory covariates	
Platelets (#/volume) in blood	TriNetX lab code: 9020
INR in plasma or blood	TriNetX lab code: 9032
